# F@#$ pain! A mini-review of the hypoalgesic effects of swearing

**DOI:** 10.3389/fpsyg.2024.1416041

**Published:** 2024-06-14

**Authors:** Carlie M. Hay, Jackson L. Sills, Julia M. Shoemake, Christopher G. Ballmann, Richard Stephens, Nicholas B. Washmuth

**Affiliations:** ^1^Department of Physical Therapy, Samford University, Birmingham, AL, United States; ^2^Department of Human Studies, University of Alabama at Birmingham, Birmingham, AL, United States; ^3^Center for Engagement in Disability Health and Rehabilitation Sciences (CEDHARS), University of Alabama at Birmingham, Birmingham, AL, United States; ^4^University of Alabama at Birmingham, University Center for Exercise Medicine (UCEM), Birmingham, AL, United States; ^5^School of Psychology, Keele University, Keele, United Kingdom

**Keywords:** swearing, cursing, pain, taboo, dosage

## Abstract

Swearing, or the use of taboo language, has been repeatedly shown to induce hypoalgesia. While reliable hypoalgesic effects have been observed across studies, the mechanisms by which swearing influences pain and the optimal dosage of swearing remain poorly understood. Plausible mechanistic rationale for swearing’s impact on pain include sympathetic response, emotion, humor, distraction, aggression, state disinhibition, psychological flow, risky behavior, and self-confidence. It remains unknown how the intensity of the swear word, speech volume, frequency, or timing influences pain modulation. While the majority of evidence demonstrates the efficacy of swearing at attenuating acute pain responses, these studies have utilized healthy populations with controlled experiments in laboratory settings. Comparatively, less is known about how laboratory findings translate practically/clinically to diverse populations, various dosages, and different pain chronicities. A greater understanding of mechanistic underpinnings and practical implications are necessary to feasibly implement swearing as a therapeutic modality to combat pain. The purpose of the following mini-review is to provide an overview of the current evidence on swearing for the reduction of pain, speculate on plausible underlying mechanisms, and discuss the potential for optimization of swearing for real-world translation. Lastly, identifying knowledge gaps to aid in directing future research will be discussed.

## Introduction

Swearing, also knowns as cursing, is broadly defined as the use of taboo words with the potential to offend (Beers Fägersten, 2012). Swearing is a near-universal feature of language (Van Lancker and Cummings, 1999) and is highly prevalent in many societies. Most of the population admits to swearing “sometimes” or “often” (Beers Fägersten, 2012), 70% of adults report frequently hearing others swear in public (Ipsos Public Affairs, 2006), most workers swear in the workplace (Williams and Uebel, 2022), and only 9% of college students indicate they have “never” encountered a professor swearing (Generous and Houser, 2019). The positive physiological, psychological, and social effects of swearing may contribute to its high prevalence. Swearing has been shown to improve physical performance (Stephens et al., 2018, 2022; Jiannine and Antonio, 2023), increase self-confidence (Stephens et al., 2022), increase humor (Beers Fägersten, 2012; Stephens et al., 2022), improve credibility (Rassin and van der Heijden, 2005), strengthen social connections (Daly et al., 2004; Stapleton, 2010; Beers Fägersten, 2012; Giffin, 2016), and improve memory and recall (MacKay et al., 2004; Jay et al., 2008). Thus, swearing appears to produce beneficial effects across various physiological and psychological domains.

Stephens et al. (2009) published the first report showing that swearing induces hypoalgesia. In this study, participants completed a cold pressor task, holding their hand in ice water for as long as possible while swearing. This study found that swearers could hold their hand in ice water for longer (increased pain tolerance). The pain modulating effect of swearing captured attention on a grand scale, with a Netflix show History of Swear Words and MythBuster’s episode “No Pain, No Gain” (MythBusters, 2010) discussing the hypoalgesic effect of swearing in detail. The notion that swearing can improve pain tolerance also got the attention of scholars. The cold-pressor task experiment has been repeated several times since 2009, with slightly different methodology, allowing for a better understanding of the hypoalgesic effects of swearing. Despite the media attention and a growing body of research, an inclusive summary of the evidence is lacking. Thus, the primary aim of this mini-review is to summarize the existing evidence on the hypoalgesic effects of swearing, including mechanistic evidence and what is currently understood related to swearing dosage. This review also aims to identify knowledge gaps to direct future research.

## Overview of swearing and pain modulation

The initial investigation into the impact of swearing on pain, to our knowledge, occurred in 2009, where Stephens et al. (2009) conducted an experiment examining the impact of swearing during a cold pressor task. Participants in this study self-selected a swear word and a non-swear word, then submerged their hand in ice water (5°C) for as long as possible while either repeating their swear word or non-swear word. This cold pressor task was chosen due to its ability to elicit pain and discomfort without tissue damage and has been determined to be ethically acceptable in adults and children (Birnie et al., 2010). The dependent variables included heart rate, perceived pain, and pain tolerance (cold pressor latency). Swearing, inherently emotional in nature, can potentially increase physiological arousal, such as heart rate. Research indicates a distinct connection between speech and its influence on autonomic control centers (Bubser and Deutch, 1998). Thus, one of the objectives of the study of Stephens et al. (2009) was to determine the effect of swearing on heart rate, a metric linked to sympathetic activation. Findings revealed that when participants repeated their self-selected swear word, cold pressor latencies were extended, indicative of pain tolerance, increased concomitantly with increases in heart rate, and a decrease in perceived pain. Importantly, the impact of swearing on cold pressor latency (pain tolerance), perceived pain, and heart rate had large effect sizes and adequate power of >0.999 (Cohen, 1988; Stephens et al., 2009). Following this cornerstone investigation, Stephens and Umland (2011) repeated the study, with findings consistent with their preview work (Stephens et al., 2009). Notably, 73% of participants kept their hand in the ice water longer while swearing, and, on average, participants held their hand in the ice water for 31 s longer while swearing. Furthermore, heart rates, again, increased more while swearing, compared to repeating a non-swear word. In line with their previous research, Stephens and Umland (2011) observed a large effect size for cold pressor latency (pain tolerance); however, contrary to their previous findings, only small effect sizes (Cohen, 1988) were noted for perceived pain and heart rate.

Philipp and Lombardo (2017) conducted a study to examine whether swearing attenuates social pain (e.g., the feeling of suffering brought on when social connections are lost or threatened). Participants completed an autobiographical writing task that asked them to complete a written reflection about a time they felt “include or accepted” or a time they felt “rejected or excluded.” Immediately after this writing task, participants repeatedly swore out loud for 2 min. Participants then completed a self-reported measure on social pain and a cold pressor task to assess physical pain sensitivity. For participants who wrote about a time they felt “rejected or excluded,” swearing significantly alleviated both social pain and physical pain sensitivity, with large effect sizes observed for both aspects. This is the first study, to our knowledge; suggesting swearing is able to attenuate social pain. While novel, these findings are not surprising since previous evidence has suggested that social pain is attenuated by other common analgesics such as marijuana (Deckman et al., 2014) and acetaminophen (DeWall et al., 2010).

Robertson et al. (2017) conducted a study involving the cold pressor task, examining whether a cross-cultural (Japanese vs. English speakers) effect exists for the hypoalgesic effects of swearing. Native English speakers repeated either “fuck” or “cup,” while native Japanese speakers repeated “kuso” (shit) or “kappu” (cup), during the cold pressor task. Swearing led to greater pain tolerance, in both native English and Japanese speakers, with medium effects sizes observed. Interestingly, even though swearing improved pain threshold (cold pressor latencies), swearing did not reduce participants’ subjective reports of pain. This suggests that swearing may increase pain tolerance across various cultural backgrounds. However, further research is needed to identify which cultures, beyond English and Japanese native speakers, experience this effect. The study of Robertson et al. (2017) included participants who were either native English or native Japanese speakers, who swore in their native language. Extensive research consistently demonstrates that multilingual individuals perceive swearing in their native language as possessing greater emotional intensity compared to swearing in a language acquired later in life (Stapleton et al., 2022). For multilingual speakers, their native language is preferred for evoking emotion when swearing. Thus, it seems reasonable to infer that the hypoalgesic effects of swearing are more pronounced in one’s native language rather than in a language acquired later in life. However, to our knowledge, no studies have been conducted to test this assumption.

Stephens and Robertson (2020) investigated the effects of repeating the conventional swear word “fuck,” a neutral word, and the two made-up swear words, “fouch” and “twizpipe,” on pain threshold and pain tolerance. Repeating “fuck” resulted in a delayed in pain onset (increased pain threshold) and an enhancement of pain tolerance (longer cold pressor latencies) compared to repeating a neutral word, “fouch,” or “twizpipe.” These statistically significant findings supporting the hypoalgesic effect of “fuck” were accompanied by a small to medium effect size. Conversely, there were null effects for “fouch” and “twizpipe,” with effect sizes falling below the smallest threshold of interest (*d* = 0.30).

More recently, Hostetter and Rascon-Powell (2022) explored whether a taboo gesture, specifically the “middle finger,” reduced the experience of pain similar to the effect of swearing. Participants submerged their non-dominant hand in the cold pressor as they repeated “fuck” or “flat,” or repeatedly extended their “middle finger” or their “index finger” with their dominant hand. Their study revealed that pain tolerance increased by engaging in a taboo act, and this effect was similar regardless of whether the participant said “fuck” or produced the “middle finger.” While both taboo acts (“fuck” and “middle finger”) increased pain tolerance, they did not impact pain threshold (onset of pain) (Hostetter and Rascon-Powell, 2022). Hostetter and Rascon-Powell (2022) did not report effect sizes or conduct power analysis. However, Jacobs et al. (2019) conducted two well-powered experiments to assess the pain modulating effect of the “middle finger” gesture. Their results showed no significant impact of taboo gestures on pain tolerance or pain perception (Jacobs et al., 2019). These inconsistent findings may stem from variations in the experimental procedures. Jacobs et al. (2019)’s study involved participants submerging their non-dominant hand in the cold pressor with the “middle finger” statically extended, while Hostetter and Rascon-Powell (2022) instructed participants to repeatedly extend their dominant hand’s “middle finger” while their non-dominant hand was in the cold pressor. The findings remain inconclusive regarding the influence of taboo gesture on pain.

Collectively, these well-powered studies consistently show that swearing effectively modulates acute pain in relatively young and healthy participants within a laboratory setting. This suggests that the hypoalgesic effects of swearing are a reliable finding. A summary of the literature exploring the hypoalgesic effects of swearing can be seen in Table 1.

**Table 1:** Reviewed studies on how swearing influences pain.

Study	Conditions	Dosage/Approach	Primary findings
Stephens et al. (2009)	Swear word, Neutral word	Swear at a constant pace and normal volume during cold pressor task.	⇑ pain tolerance, ⇑ heart rate, ⇓ perceived pain
Stephens and Umland (2011)	Swear word, Neutral word	Swear at a constant pace and normal volume during cold pressor task.	⇑ pain tolerance, ⇑ heart rate, ⇔ perceived pain
Philipp and Lombardo (2017)	Swear word, Neutral word	Swear for 2 min repeatedly prior to cold pressor task.	⇓ social pain, ⇓ perceived pain
Robertson et al. (2017)	Swear word, Neutral word, Native English, Native Japanese	Swear at a constant pace and normal volume during cold pressor task.	⇑ pain tolerance, ⇔ subjective reports of pain
Stephens and Robertson (2020)	Swear word, Neutral word, Made up swear words “fouch” and “twizpipe”	Swear every 3 s and normal volume during cold pressor task.	⇑ pain threshold, ⇑ pain tolerance, ⇑ emotion, ⇑ humor, ⇑ distraction, ⇔ heart rate
Hostetter and Rascon-Powell (2022)	Swear word, Neutral word	Swear every second during cold pressor task.	⇑ pain tolerance, ⇔ aggressive thoughts

Conditions, swearing dosage, physical task, and primary findings from each investigation are presented. ⇑ indicates an increase, ⇓ indicated a decrease, and ⇔ indicates no change.

## Potential mechanisms of pain modulation

### Physiological

**Figure 1:**
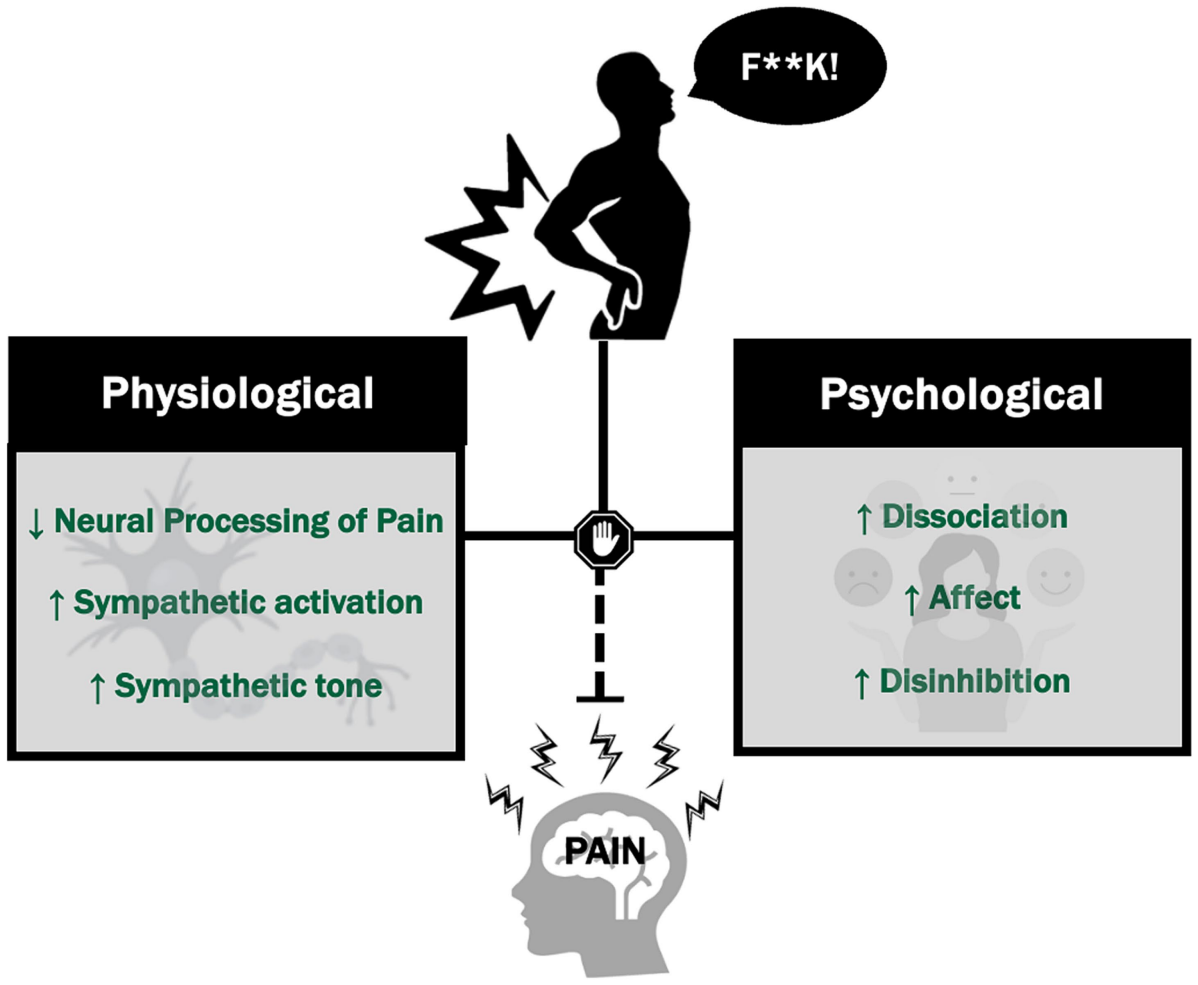
Potential mechanisms for swearing-induced pain modulation.

The mechanistic underpinnings of swearing-induced hypoalgesia remain to be fully elucidated. The initial proposed mechanism behind the hypoalgesic effects of swearing revolved around an increase in heart rate, aligning with the activation of the fight or flight response. Stephens et al. (2009) and Stephens and Umland (2011) observed enhanced pain tolerances and elevated heart rates among participants who swore, indicating sympathetic activation (associated with the fight or flight response) as a potential mechanism by which swearing modulates pain. However, subsequent research, including studies by Hostetter and Rascon-Powell (2022) and Stephens and Robertson (2020), implemented the cold pressor task while monitoring heart rate, revealed inconsistent outcomes. Despite observing a pain modulating effect of swearing, these studies did not detect any differences in heart rate between swearing and non-swearing conditions. These conflicting findings regarding heart rate responses present challenges in interpretation. It is plausible that the heart rate measures utilized in these studies were insufficiently sensitive to capture subtle changes in sympathetic activation, or alternatively, the mechanism underlying swearing’s impact on pain may be something other than sympathetic activation (Figure 1).

A neuromodulatory effect of swearing has also been described, which can alter pain responses. Neuroimaging investigations have reported greater amygdala and pre-frontal cortex (PFC) activation when speaking taboo vs. neutral words (Chun et al., 2015). Indeed, amygdala activation is characteristic of heightened pain responses and is present clinically in chronic pain and experimental protocols (Simons et al., 2014). PFC connections, known to be important in speech processing (Hertrich et al., 2021), to the amygdala have also been shown to alter perceptions of stressful and painful stimuli altering behavioral responses (Liu et al., 2020). Thus, it is plausible that swearing alters PFC-amygdala crosstalk and/or activation whereby pain perception is attenuated, and behavior altered. However, this is mostly speculative at the moment and further swearing and neuroimaging investigations are needed to understand mechanistic determinants.

### Psychological

It has also been hypothesized that the beneficial effects of swearing on pain may be attributed to factors such as emotion, humor, and distraction (Stephens and Robertson, 2020). It has been suggested that the emotion-provoking aspect of swearing increases autonomic arousal (Stephens and Allsop, 2012). Another theory posits that swearing reduces pain through attention modulation (Wiech et al., 2008). Swearing may distract attention away from a painful stimulus; thereby reduce perceived pain (Edwards et al., 2009). A property of swearing that may effectively distract from pain is its potential for humor (Stephens and Robertson, 2020). For example, the word “fuck” is rated among the top 1% of the funniest words (Engelthaler and Hills, 2018) and may modulate pain via humor-induced distraction (Stephens and Robertson, 2020). Stephens and Robertson (2020) investigated the impact of different words, including “fuck,” a self-selected neutral word, and two made-up swear word, “fouch” and “twizpipe,” on performance during the cold pressor task. While repeating “fuck,” “fouch,” and “twizpipe” correlated with ratings of emotions, humor, and distraction among participants, none of these factors—emotion, humor, or distraction—were predictive of pain tolerance. These results suggest that the pain modulating effects of swearing are not driven by emotional, humorous, or distracting aspects.

The hypoalgesic effects of swearing has been proposed as being related to aggression (Stephens and Zile, 2017). Swearing often co-occurs with aggressive feelings, and high frequency of swearing is associated with a hostile personality (Jay, 2009). However, Hostetter and Rascon-Powell (2022) found even though participants’ pain tolerances increased while swearing, swearing did not increase participants’ aggressive thoughts. The swearing in this study may have caused a catharsis effect (decrease aggression) (Vingerhoets et al., 2013), or it may be argued that the Word Completion Task (Anderson et al., 2003) used in the study to measure aggressive feelings was not sensitive enough to detect changes in aggression. Nevertheless, there are inconsistent findings related to aggression as a mechanism.

It is plausible that swearing’s capacity to modulate pain may stem from a combination of mechanisms or that these mechanisms vary based on the unique biopsychosocial characteristics of each individual. For instance, swearing may modulate pain by leveraging the combined mechanisms of increased sympathetic activation and distraction driven by humor. Related to the biopsychosocial characteristics of the individual, it is plausible that personality traits may impact when or how swearing mediates the effects of pain, as it has been shown that those with “agreeableness” personality traits tend to swear less frequently in their everyday life (Mehl et al., 2006). Additionally, the notion has been posited that factors such as state disinhibition, psychological flow, engagement in risky behavior, and enhanced self-confidence could be linked to the beneficial effects of swearing (Stephens et al., 2022).

## Swearing dosage

The swearing dosages used across these studies have been consistent. Among the six studies investigating the effects of swearing on pain modulation, all have involved participants verbally swearing at a steady pace and volume (Stephens et al., 2009; Stephens and Umland, 2011; Philipp and Lombardo, 2017; Robertson et al., 2017; Stephens and Robertson, 2020; Hostetter and Rascon-Powell, 2022). In five out of the six studies, participants were instructed to repeat a swear word during the cold pressor task (Stephens et al., 2009; Stephens and Umland, 2011; Robertson et al., 2017; Hostetter and Rascon-Powell, 2022), while Philipp and Lombardo (2017) had participant swear repeatedly for 2 min before the task. Regarding swearing frequency, Hostetter and Rascon-Powell (2022) had participants swear once per second, while Stephens and Robertson (2020) instructed participants to swear once every 3 s, with other studies indicating participants repeated the swear word at a consistent pace (Stephens et al., 2009; Stephens and Umland, 2011; Philipp and Lombardo, 2017; Robertson et al., 2017). Three studies allowed participants to self-select their swear word (Stephens et al., 2009; Stephens and Umland, 2011; Philipp and Lombardo, 2017), while three studies asked the participants to repeat “fuck” (Robertson et al., 2017; Stephens and Robertson, 2020; Hostetter and Rascon-Powell, 2022). The consistent application of swearing dosages across these studies likely contributed to the observed similarities in pain modulation effects.

Concerning the dosage of swearing, individual differences in daily swearing frequency may impact the hypoalgesic effects of swearing. Stephens and Umland (2011) revealed a habituation phenomenon regarding swearing’s hypoalgesic effects, wherein individuals who swear more frequently in their daily lives exhibited a diminished hypoalgesic response when they swore. This finding is consistent with an experiment conducted by Philipp and Lombardo (2017), which found that swearing for 2 min reduced feelings of pain; however, individuals who swore less often in their daily lives experienced a more pronounced hypoalgesic effect compared to those who swore more often. This suggests that excessive use of swearing in everyday situations diminishes its efficacy as a short-term intervention for reducing the perception of pain.

## Future research

Swearing has consistently demonstrated its ability to modulate acute pain across six studies, suggesting a reliable effect. However, these investigations were conducted in controlled laboratory settings, predominantly involving college-aged participants, and included acute pain induced by the cold pressor task. There are many methodological challenges in human subject’s research, particularly in relation to the study of swearing (Stapleton, 2020). The absence of blinding among research participants, who are aware of when they are swearing, is another significant issue. Furthermore, given the considerable media coverage surrounding this topic, participants may infer the hypotheses being tested in these studies. The age, gender, and race of the research investigators collecting the data may add confounding and contextual effects of the swearing (Beers Fägersten, 2012). It has also been noted that college students, which are the majority of the participants in the experiments examining the impact of swearing on pain, have expressed concerns about swearing in front of a professor during experiments involving swearing (Jiannine and Antonio, 2023). Consequently, it is plausible that participant responses are influenced by artifact explanations. Future research should expand its scope to explore the effects of swearing on pain modulation in naturalistic (real-world) settings, on diverse populations, and include various forms of pain, such as sub-acute or chronic conditions induced by stimuli such as heat or electrical shocks.

Further mechanistic research is warranted to elucidate the underlying reasons for swearing’s effects, offering insights into optimizing hypoalgesic outcomes. It is imperative for studies to not only investigate whether swearing works but also examine how it works. Future research should continue examining plausible mechanisms, including sympathetic activation, emotion, humor, distraction, state disinhibition, psychological flow, risky behavior, and self-confidence. Interdisciplinary collaborations for mechanistic research may yield valuable results, particularly if there are combined mechanisms at play or if patient profiles, such as biopsychosocial characteristics, influence the effects of swearing on hypoalgesia.

Moreover, future studies should explore the impact of different swearing dosages on pain modulation. Dosage appears to be an important factor for interventions aimed at modulating pain. Ice is commonly used to manage pain and there appears to be an optimal icing dosage for the management of acute pain (Bleakly et al., 2004). Conversely, nonsteroidal anti-inflammatory drugs, another common treatment for acute pain, appears to have similar analgesic effects across a variety of dosages (Motov et al., 2019). Currently, it remains unknown how different dosages of swearing, including the intensity of the swear word, speech volume, frequency, or timing of swearing, impacts pain modulation. Investigating whether variations in the intensity of the swear word (e.g., “damn” vs. “fuck”), speech volume (e.g., whisper vs. normal speech volume vs. shouting), frequency (e.g., once per second vs. once every 30 s vs. once per minute), or timing (e.g., swearing before pain stimulus vs. swearing during pain stimulus vs. swearing before and during pain stimulus) influences pain modulation. Additionally, the impact of being sworn at compared to swearing oneself on pain modulation has yet to be explored. For instance, the effects of someone telling an individual to “ignore that fucking pain” compared with the individual swearing themselves is currently unknown.

For ethical reasons, using methodology to control for confounding and contextual effects may not be possible, which leaves researchers with challenging methodological issues when designing experiments to increase the understanding of the effects of swearing on pain. However, continuous research efforts are necessary to gain a deeper understanding of swearing’s impact on the experience of pain. Empirical investigations incorporating variables related to naturalistic settings, participant diversity, various dosages, and different pain chronicities will enhance our understanding of the complexities of swearing and its relationship with pain.

## Conclusion

In summary, swearing has shown consistent effectiveness in modulating acute pain across multiple studies, indicating a reliable effect. However, the majority of these studies were conducted in controlled laboratory settings using the cold pressor task to induce pain. Future research should broaden its scope to investigate swearing’s impact on pain modulation in real-world scenarios, diverse populations, and various types of pain. Mechanistic research and exploring different swearing dosages is also crucial for a comprehensive understanding of swearing’s effect on pain. Once such evidence becomes available, it will foster a deeper understanding of when, how, and if swearing can be used to effectively modulate pain.

## Author contributions

CH: Conceptualization, Writing – review & editing. JSi: Conceptualization, Writing – review & editing. JSh: Conceptualization, Writing – review & editing. CB: Conceptualization, Writing – review & editing. RS: Data curation, Writing – review & editing. NW: Project administration, Supervision, Writing – original draft, Writing – review & editing.
